# Violence sexuelle sur mineurs dans une population tunisienne: étude de 53 expertises psychiatriques

**DOI:** 10.11604/pamj.2023.44.157.39367

**Published:** 2023-04-04

**Authors:** Khaoula Khemakhem, Jaweher Boudabous, Khadija Chiha, Imen Hadjkacem, Hela Ayadi, Yousr Moalla

**Affiliations:** 1 Service de Psychiatrie de l´Enfant et de l´Adolescent de Sfax, Centre Hospitalier Universitaire Hédi Chaker de Sfax, Faculté de Médecine de Sfax, Sfax, Tunisie

**Keywords:** Mineur, violence sexuelle, stress post-traumatique, Minor, sexual abuse, post-traumatic stress

## Abstract

En Tunisie, le nombre déclaré d'enfants victimes d'abus sexuels a connu une évolution ces dernières années. Les psychiatres d´enfants sont sollicités pour effectuer des expertises et pour des soins. Les objectifs de notre travail étaient de décrire le profil socio-familial et clinique des enfants victimes de violence sexuelle et de décrire les données concernant les circonstances et la nature de l´abus sexuel. Il s´agit d´une étude rétrospective descriptive et analytique, portant sur des cas d´enfants, examinés par des pédopsychiatres exerçant à l´hôpital Hédi Chaker de Sfax dans le cadre d´expertise psychiatrique suite à une violence sexuelle. Durant la période allant de janvier 2011 à octobre 2017. Le nombre d´enfants vus en expertise pour violence sexuelle durant la période d´étude était de 53. Les victimes étaient des filles dans 64,2% des cas, d´âge moyen 10 ans, des élèves du primaire dans 46,2% des cas. Un conflit intrafamilial était retrouvé dans 16,6% des cas. L´abus était intrafamilial dans 25% des cas, les attouchements et la pénétration anale étaient les types de violence les plus fréquents observés respectivement dans 63,5% des cas et 26,9% des cas. Plus que la moitié des enfants victimes présentait un trouble psychiatrique, il s´agissait d´un état de stress post traumatique dans 32,7 % des cas. A travers cette étude, la violence sexuelle chez les mineurs reste un problème complexe avec de lourdes conséquences chez l´enfant victime. Des mesures sont à entreprendre pour garantir à l´enfant, une intervention adéquate.

## Abstract (en)

In Tunis, the reported cases of children who are victims of sexual abuse have increased in recent years. Child psychiatrists are asked to perform a psychiatric evaluation and treat patients. The purpose of this study was to describe the socio-familial and clinical profile of children who were victims of sexual violence and to describe data concerning the circumstances and nature of sexual abuse. We conducted a descriptive and analytic, retrospective study of children examined by child psychiatrists practising at the Hédi CHAKER hospital in Sfax following sexual violence. The study covered the period from January 2011 to October 2017. Child psychiatrists evaluated 53 child sexual abuse cases during the study period. The victims were girls in 64.2% of cases, with an average age of 10 years and primary school students in 46.2% of cases. Intra-family conflict was reported in 16.6% of cases. Intra-familial abuse was reported in 25% of cases, touching and anal penetration were the most frequent types of violence observed (63.5% of cases and 26.9% of cases respectively). More than half of children who were victims had a psychiatric disorder (post-traumatic stress in 32.7% of cases). This study shows that child sexual abuse is a complex problem with serious consequences. Measures must be taken to ensure children have adequate treatment.

## Introduction

La violence sexuelle commise à l´encontre des enfants a été longtemps considérée comme un sujet tabou et dont l´existence a été niée. La reconnaissance internationale des droits de l´enfant tels que stipulés dans la convention internationale des droits de l´enfant, ratifiée par la Tunisie dès 1994, a permis une prise de conscience de la gravité des différentes formes de maltraitance et abus subis par les mineurs [1].

Selon l´Organisation mondiale de la Santé (OMS), l´exploitation sexuelle d´un enfant implique qu´il est victime d´un adulte ou d´une personne sensiblement plus âgée que lui, aux fins de la satisfaction sexuelle de celui-ci. Elle précise qu´il s'agit de toute activité sexuelle que l'enfant n'est pas pleinement en mesure de comprendre, à laquelle il ne peut consentir en connaissance de cause ou pour laquelle il n'est pas préparé du point de vue de son développement, ou encore qui viole les lois ou les tabous sociaux [2]. Les différentes revues de littérature consacrées aux violences sexuelles rapportent que 15 à 30% des filles et que 5 à 15% des garçons sont victimes de violences sexuelles avant 18 ans [3]. Néanmoins, l'OMS s'accorde à dire que ces violences sont sous-estimées. Les violences sexuelles connues ne seraient ainsi que la partie émergée de l'iceberg [4].

En Tunisie, le nombre d'enfants victimes d'abus sexuels a triplé de 2013 à 2016. Il est passé de 261 à 601, en se référant aux cas signalés auprès des délégations à la protection de l'enfance. Le nombre réel serait plus grand, selon les spécialistes, travaillant sur ce sujet [5]. Des professionnels sont amenés à intervenir face aux enfants victimes de violence sexuelle notamment les psychiatres d´enfants et d´adolescents qui sont sollicités aussi bien pour effectuer des expertises que pour des soins. Dans la présente étude, nous nous sommes intéressés à l´étude de la violence sexuelle chez les mineurs adressés au service de psychiatrie de l´enfant et de l´adolescent pour expertise psychiatrique.

Les objectifs de notre travail étaient de décrire le profil socio-familial et clinique des enfants victimes de violence sexuelle, de décrire les données concernant les circonstances et la nature de la violence sexuelle.

## Méthodes

**Type de l´étude:** il s´agit d´une étude rétrospective descriptive et analytique.

**Cadre de l'étude et population:** nous avons inclus tous les enfants examinés au service de pédopsychiatrie de l´hôpital Hédi Chaker de Sfax durant la période allant de janvier 2011 à octobre 2017 dans le cadre d´une expertise psychiatrique pour violence sexuelle.

**Ressource de données et mesure:** pour réaliser les objectifs de l´étude, nous nous sommes référés au dossier d´expertise. Pour chaque cas, nous avons transcrit sur une fiche les renseignements recueillis à partir des dossiers, les données épidémiologiques de la victime, des informations concernant l´auteur de l´abus (son sexe, son lien de parenté avec l´enfant), la violence sexuelle (nature, fréquence, autre type de violence associée) et la symptomatologie clinique présentée par le mineur. Les diagnostics cliniques ont été retenus selon les critères du DSM-IV-TR [6].

### Analyse des données

La saisie des données et l'analyse statistique ont été réalisées à l'aide du logiciel SPSS 20.0. Pour l'étude descriptive, les variables qualitatives ont été rapportées en pourcentage, alors que les variables quantitatives ont été rapportées en moyenne avec leurs écarts types. Pour l'étude analytique, les comparaisons ont été effectuées par le test de Chi-deux (χ^2^) pour les données qualitatives, et par le test t de Student pour les données quantitatives. Le seuil de significativité a été fixé à 5%.

## Résultats

**Données relatives à la victime:** durant la période de notre étude, 53 mineurs victimes de violence sexuelle ont été colligés. Les données relatives à ces victimes sont présentées dans le Tableau 1.

**Tableau 1:** données liées à la victime

Données		Pourcentage (%)
**Sexe**	filles	64,2
	garcons	35,8
**Age moyen**	10 ans ±3,9	
**Age < 4 ans**	4	9,4
**Pubertés**		28.8
**Zone de résidence**	urbaine	61.5
	semi urbaine	15,4
	rurale	19, 2
**Scolarité**	non encore ou préparatoire	19,2
	primaire	46,2
	collégiens et lycéens	15,3
	en arrêt de scoalrité	9,5
**Niveau sociéconomique**	moyen	53,8
	bon	17,3
	bas	15,4
	non précisé	13,5
**Structure de la famille et dynamique familiale**	monoparentale	16,9
	conflit intrafamilal	16.6
	violence intrafamiliale	9.6
**Antécédents personnels médico-psychiatriques**		13.3
	handicap intellectuel léger	1,9
	surdi-mutité	1,9
	bégaiement et difficultés scolaires	1,9
	trouble déficit de l’attention/hyperactivité	1,9
	suspicion d’abus	1,9
	méningite	1,9
	obésité morbide	1,9
**Antécédents familiaux**	Suivi psychiatrique chez la mère	1,9
	Incarcération du père	3,8

**Données liées à la violence sexuelle:** les données concernant l´auteur de la violence, la nature des sévices subis par l´enfant sont résumées dans les Tableau 2 et Tableau 3.

**Tableau 2:** caractéristiques de l´agresseur

Caractéristiques		Pourcentage (%)
Nombre	Une seule personne	91,7
	Deux personnes différentes	8,3
Sexe	Masculin	98,1
	Féminin	1,9
Age	<20 ans	5 ,8
	[20-35 ans]	26,4
	[36-50 ans]	35,5
	>50 ans	32,4
Type de l’abus et lien entre la victime et l’auteur de l’abus	Intra familial	25
	Père	7,6
	Beau-père	1,9
	Grand-père	1,9
	Oncle	5,7
	Cousin	7,6
	Extra familial	75
	Etranger non connu	26,6
	Voisin	17,1
	Enseignant	15,2
	Ami	5,7
	Vendeur	7,6
	Patron	3,8

**Tableau 3:** nature et fréquence des sévices subis par l´enfant

Nature et fréquence des sévices	Pourcentage (%)
Violence sexuelle	100
Exposition à la pornographie	1.9
Exhibitionnisme	23.1
Attouchements	63.5
Fellation	9.6
Pénétration anale	26.9
Pénétration vaginale	5.8
Violence physique	46,2
Violence morale	50
Unique	51,9
Répété	48,1

**Symptomatologie clinique:** la symptomatologie clinique retrouvée à l´entretien est rapportée dans le Tableau 4. Un même enfant pouvant présenter plusieurs symptômes à la fois. En fin d´expertise un diagnostic nosographique a été avancé dans 54,16% des cas, il s´agissait d´un état de stress post traumatique (ESPT) dans 32,7% des cas, d´un état de stress aigu (ESA) dans 19,2% des cas, de dépression dans 5,8% cas et d´une comorbidité ESPT et dépression dans 1,9 % des cas. Dans le reste des cas, l´expertise a conclu à des manifestations anxieuses, difficultés psychologiques, éléments dépressifs sans diagnostic nosographique et dans 13,5% des cas l´examen psychiatrique était normal.

**Tableau 4:** symptomatologie clinique chez les enfants examinés

Symptômes cliniques	Pourcentage (%)
Trouble du sommeil	71.2%
Troubles sphinctériens/énurésie	5.8%
Somatisation	15.4%
Anorexie	19.2%
Irritabilité	44.2%
Troubles de la concentration	46.2%
Difficultés scolaires	38.5%
Pleurs	34.6%
Troubles du comportement externalisé instabilité motrice/ agitation/agressivité	21.2%
Troubles sexuels	7.7%
Phobies	76.9%
Evitement	67.3%
Reviviscence	76.9%
Retrait	32.7%
Conduites régressives	28.8%
Aucun	13.5%

**Etude analytique:** dans un deuxième temps de l´étude, des corrélations ont été recherchées entre le genre de la victime, l´âge de la victime (< 12 ans, ≥ 12 ans) et le type d´abus (intra ou extrafamilial) avec la nature de la violence sexuelle et la symptomatologie clinique.

**Selon le genre:** le sexe féminin était corrélé au retrait (p=0.05) et aux somatisations (p=0.014) alors que le genre masculin était corrélé aux conduites régressives (p=0.05).

**Selon l´âge:** la victime âgée de plus de 12 ans, était plus exposée à la pénétration vaginale (p=0.02). Pour la symptomatologie clinique présentée, après l´âge de 12 ans, les victimes présentaient plus de symptômes de retrait (p=0.05), de pleurs (p=o), et d´anorexie (p=0.005). Alors qu’avant l´âge de 12 ans, c´était plutôt les troubles du comportement qui caractérisaient la symptomatologie clinique (p= 0.018).

**Selon le type d´abus intra ou extrafamilial:** l´abus intrafamilial était corrélé à des sévices répétés (p=0.04), à la pénétration anale (p=0.045) et à la pénétration vaginale (p =0.017). La symptomatologie clinique en cas d´abus intrafamilial était corrélé aux troubles sphinctériens (p=0.01), troubles des conduites sexuelles (p=0.017) et conduites régressives (p =0.05).

## Discussion

### Données liées à la victime

Dans la présente étude, les victimes d´abus sexuel étaient des filles dans 63.5% des cas et des garçons dans 36.5% des cas, l´âge moyen des victimes était de 10 ans ± 3,9, cinq enfants étaient âgés de 4 ans ou moins, 15 victimes étaient pubères au moment de l´agression. Les données de la littérature s´accordent sur la prépondérance féminine des violences sexuelles sur mineurs [7,8]. Plusieurs études internationales concluent à des taux de 1,5 à 3 fois supérieurs chez les filles [9,10].

Une étude rétrospective réalisée à partir des registres juridiques des affaires d´agressions sexuelles commises dans une région du Centre-Est tunisien jugées entre 1993 et 1998 a révélé que 629 affaires d´agressions sexuelles ont été recensées, correspondant à une incidence de 14,7 victimes pour 100 000 habitants par an, 81% des victimes étaient mineures et 58% étaient de sexe féminin [11]. A l´inverse, dans une étude tunisienne de 28 expertises d´abus sexuel sur mineurs, une prédominance des victimes de sexe masculin a été rapportée. Les auteurs ont expliqué ce constat par le fait que les garçons victimes seraient plus souvent amenés à des expertises psychiatriques à la recherche d´éventuelles répercussions psychologiques de l´abus alors que les filles bénéficieraient plutôt d´une expertise médico-légale afin de s´assurer de leur virginité [11]. Concernant l´âge de la victime, une étude tunisienne réalisée à Monastir sur les mineurs victimes d´abus sexuel a trouvé le même résultat que notre étude [12].

Selon Messerchmitt, il existe deux pics de fréquence de l´abus sexuel chez les enfants vers 6 ans et vers 13-14 ans [13]. Une étude canadienne a comparé, durant 3 ans, les différences des caractéristiques des abus sexuels intrafamiliaux et extrafamiliaux sur mineurs. Il en ressort que les enfants victimes d´abus sexuels intrafamiliaux sont plus jeunes que les victimes d´abus extrafamiliaux. L´âge moyen des garçons et filles victimes d´abus sexuels intrafamiliaux est respectivement de 5,3 ans et 7,4 ans [14]. Dans la présente étude, parmi les victimes d´abus sexuel, 17,5% des cas avaient des antécédents médico-psychiatriques dont deux cas d´handicap. Le handicap de l´enfant a été admis comme facteur de risque, augmentant la vulnérabilité de l´enfant à être victime de sévices sexuels [15].

Quant aux données géographiques, nous avons relevé que 61,5% des victimes vivaient dans un milieu urbain. Ce chiffre peut être expliqué par la difficulté d´accessibilité des régions rurales à la justice et les longues procédures contraignantes que ces habitants peuvent rencontrer d´autant plus que ce sujet est encore considéré comme tabou dans ces régions. Dans la présente étude, la famille des enfants victimes d´abus avait un niveau socio-économique moyen dans 53,8% des cas, bon dans 17,3% des cas et bas dans 15.4% des cas. Toutes les études s´accordent à dire que les violences sexuelles ne touchent pas une communauté ou un groupe socio-économique spécifique. Tous les enfants issus de différents milieux peuvent être touchés [12]. Dans la présente étude, un conflit intra-familial ou de la violence intrafamiliale ont été retrouvés dans respectivement 16,6% et 9,6% des familles des victimes. Le père était incarcéré dans deux cas. Certains groupes d´enfant sont plus exposés à la violence sexuelle que d´autres. Le risque est plus important pour les enfants grandissant au sein d´une famille dans laquelle des actes de violence ou d´abandon moral ont déjà été commis. C´est également le cas des enfants qui vivent sous le même toit ou dans l´entourage d´hommes eux-mêmes victimes dans leur enfance de violence sexuelle [12].

### Données liées à la violence sexuelle

Dans cette étude, l´agresseur était un homme dans 97% des cas, âgé de moins de 20 ans dans 5,8% des cas, entre 20 ans et 35 ans dans 26,4% des cas, entre 36 ans et 50 ans dans 35,4% des cas et de plus de 50 ans dans 32,4% des cas. La majorité des auteurs d'abus sexuels commis sur les enfants sont des hommes [11]. Plusieurs auteurs s'entendent cependant pour reconnaître que le nombre d'abus sexuels commis par des femmes peut être sous-estimé par les données actuelles. Il est fort possible, en effet, que les abus commis par des femmes, surtout sur des garçons soient un sujet plus tabou. Néanmoins, aucun indice sérieux ne permet de supposer qu'une proportion beaucoup plus élevée de femmes, que celle rapportée par les études actuelles, commettraient des abus sexuels envers les enfants [11].

Les études consultées concluent que la majorité des auteurs d'abus sexuels sur les enfants ont un âge variant entre 35 et 40 ans. De nombreux abuseurs sont de jeunes adultes ou des adolescents. Relativement peu d'abuseurs sont des personnes âgées [11,12]. Dans notre étude, l´agresseur était un membre de la famille dans 25% des cas et un étranger connu par l´enfant dans 48% des cas. Ceci concorde avec les résultats d´une étude menée sur les agressions sexuelles chez l´enfant du grand Tunis menée au service de médecine légale de l´hôpital Charles-Nicolle de Tunis où 61% des agresseurs étaient des étrangers. A l´inverse, dans une autre étude tunisienne réalisée au service de pédopsychiatrie à Razi, il s´agit d´un abus intra-familial dans 93% des cas et d´un abus extra-familial dans 7% des cas (un directeur d´école dans un cas et un instituteur dans l´autre) [11]. Dans une autre étude tunisienne réalisée en consultation de pédopsychiatrie sur des enfants victimes de violences sexuelles, dans 37,3% des cas, l´agresseur était un membre de la famille. Le père était l´abuseur présumé dans la moitié des cas intrafamiliaux. En cas d´agressions extra-familiales, l´abuseur était connu par la victime et/ou leurs familles dans 48,66% [16].

Dans la littérature internationale, il est rapporté que l´abus sexuel est intrafamilial dans 75 à 78% des cas et extrafamilial dans 22 à 25% des cas. Dans ces derniers cas, les agresseurs sont souvent des proches de la famille ou des personnes ayant autorité sur l´enfant [7,8]. En fait les chiffres retrouvés dans notre travail, pourraient ne pas refléter la réalité, en effet, ce type d´abus peut être caché pour préserver l´équilibre familial et il est fortement réprimé dans notre société. Cette faible représentativité de l´aspect incestueux avait été signalée par plusieurs auteurs africains [17,18].

Dans la présente étude, les sévices subis par les enfants étaient à type d´attouchements dans 63.5% des cas, de fellation dans 9,6% des cas, d´exhibitionnisme dans 23,1 % des cas, et d´une pénétration anale dans 26,9% des cas et vaginale dans 5.8 % des cas. L´étude tunisienne réalisée à Monastir a trouvé les mêmes résultats [12]. La pénétration indépendamment de sa nature est accompagnée de forçage, de brutalité, de gestes plus ou moins violents. Dans tous les cas, l´acte de pénétration correspond à un viol, une invasion, une perforation, effraction de l´enveloppe intime de l´enfant. Les enfants étaient victimes d´un abus répété dans 45,1% des cas. En accord avec nos résultats, l´étude tunisienne réalisée à Monastir a trouvé que l'agression était unique dans 47% des cas et répétée dans 44% des cas [12]. Plus un abus sexuel s´installe comme mode interactif entre l´enfant et l´adulte et plus il dure plus il se chronicise et risque d´enkyster les symptômes chez la victime.

L´abus sexuel était associé à un autre type de violence dans 63,5% cas. Il s´agissait de violence physique dans 46,2% des cas et de violence morale dans 50% des cas. L´utilisation de la violence physique et/ou morale au cours de l´abus sexuel varie largement en fonction de l´âge de la victime. Avec les tout-petits, plutôt que rechercher à faire peur, l´abuseur le fait de façon « soft », en les séduisant, en leur présentant l´abus comme un jeu. L´agresseur essaie ainsi également d´imposer le secret. Par contre, la violence physique et morale, associée à l´abus est plus fréquente quand les enfants sont plus âgés [11].

### Symptomatologie clinique

Dans la présente étude, la symptomatologie clinique relevée au cours des expertises à travers l´examen de l´enfant ou l´histoire des parents était variable et polymorphe. Les troubles liés à un traumatisme sont fréquents, variés et difficiles à déterminer. Quand ils existent, ils informent sur l´état de stress et de souffrance manifestés par l´enfant [19]. Rappelons que chaque enfant abusé est particulier et unique et que mis à part la prise sur les faits ou lorsque les lésions organiques sont évidentes, la plupart des signes ne sont pas pathognomoniques d´une agression sexuelle. Il n´existe pas de modèle linéaire de cause à effet qui reprendrait tous les symptômes et qu´il suffirait d´appliquer comme simple grille de décodage [20]. Cette psychopathologie péri traumatique complexe résulte du développement de l´enfant, des remaniements identitaires et identificatoires de la puberté, de l´économie psychique intrafamiliale[19,20].

En effet, la majorité des enfants et certainement les plus jeunes ne peuvent exprimer leur souffrance par la parole. Parfois leur seul moyen d´expression est leurs corps [19]. Leur mal-être peut se manifester par différents symptômes : les troubles somatiques, les troubles sphinctériens, les avatars de la sphère alimentaire, les perturbations du sommeil. Mais aussi par des troubles du comportement autant l´inhibition, la tristesse, le manque de confiance en soi, les plaintes dépressives, l´isolement à l´opposé, l´agitation, l´agressivité, les passages à l´acte sont des points d´appel qui peuvent rentrer dans une problématique de maltraitance sexuelle. On retrouvera aussi les troubles d´apprentissage, de concentration et les chutes de rendement scolaire et les troubles d´ordre sexuel, en effet, l´enfant abusé peut manifester des comportements sexuels en relation avec une expérience prématurée de stimulation à ce niveau. Les troubles engagent tant l´expression du corps que le langage oral. Cela peut aller des provocations sexuelles, en passant par des conduites inadaptées ou exagérées jusqu´aux agressions sexuelles [19].

La haute autorité de santé spécifie les signes d'alerte spécifiques ou modes d'expression pour les adolescents: tentatives de suicide, fugues, conduites à risque, conduites d'addictions précoces, tabagisme-alcoolisation et/ou toxicomanie, comportement alimentaire compulsif, actes de violence envers les autres, automutilations dont les scarifications, demande précoce de contraception, interruption volontaire de grossesse isolée ou à répétition, changements fréquents de partenaires, actes de prostitution [21]. Chez l´enfant et l´adolescent, la prudence doit être de mise devant une symptomatologie atypique faite d´agressivité, de troubles du comportement, de troubles des apprentissages, d´exhibition sexuelle et cela même chez un enfant que l´on croit à l´abri. Dans notre étude, chez 16,7 % des d´enfants expertisés l´examen était normal. La résilience est cette capacité à pouvoir « rebondir » malgré les traumatismes.

D´après Delage et Cyrulink, ce concept se développe autour de deux axes principaux: un axe intrapsychique (capacité de représentation, de construction d´un imaginaire, capacité cognitive) et un axe relationnel [22]. Dans la présente étude, en fin d´expertise un diagnostic a été avancé dans 54,16% des cas, en se référant au DSM-IV-TR, il s´agissait d´un ESPT dans 29,2% des cas, d´un ESA dans 20.8% des cas, de dépression dans 4,16% cas et d´une comorbidité ESPT et dépression dans 2% des cas. Dans le reste des cas, l´expertise a conclu à des manifestations anxieuses, difficultés psychologiques, éléments dépressifs sans diagnostic nosographique.

Selon le DSM-IV-TR, plusieurs catégories diagnostiques peuvent s´observer après un traumatisme mais deux troubles sont spécifiques des troubles post-traumatiques: l´état de stress post-traumatique et l´état de stress aigu [6]. La représentation clinique de l´ESPT varie en fonction de l´âge des enfants. Selon Schwarz et Perry, la symptomatologie s´enrichit avec l´âge de l´enfant. Entre 2 et 6 ans, on retrouve les somatisations, les phénomènes d´évitement, la tristesse, les angoisses de séparation et des comportements régressifs, parfois des comportements de retrait, de mutisme ou de conduites agressives. Entre 6 et 12 ans: on constate d´avantage de troubles anxieux, d´affects dépressifs, de l´inhibition, de phobies, et de difficultés d´apprentissage [23,24]. Pour une exposition traumatique en général le risque que s'installe un état de stress post-traumatique est de 24%, après des violences sexuelles dans l´enfance un état de stress post-traumatique est retrouvé dans 87% des cas. Dans les cas de violences sexuelles incestueuses dans l´enfance, ce taux peut même atteindre 100% [25].

En post-traumatique, on peut trouver aussi des diagnostics comme une dépression, un trouble anxieux comme une anxiété généralisée, un trouble obsessionnel compulsif ou une anxiété de séparation, un trouble de l´adaptation, un trouble psychotique bref avec facteur de stress marqué, des décompensations de pathologies psychiatriques antérieures [26]. Ces troubles peuvent exposer à des nouveaux faits traumatiques, perdurer à l'âge adulte et entraîner des difficultés globales, notamment dans les futurs rôles parentaux. De plus en plus de recherches révèlent l'impact du psychotraumatisme sur le fonctionnement neurobiologique, notamment sur les structures hypothalamo-hypophyso-surrénaliennes [27,28]. La limite de cette étude comprend le caractère rétrospectif de l´étude se basant sur des dossiers d´expertises, des données observées par l´expert pouvant ainsi ne pas figurer dans le dossier.

## Conclusion

La question de la violence sexuelle chez les mineurs reste un problème complexe. Des agressions de nature différente, perpétuées à l´enfant par un membre de la famille ou par une personne étrangère parfois répétées, l'impact émotionnel et psychologique est important avec des expressions cliniques variables. Les efforts déployés jusque-là restent insuffisants et des mesures sont à entreprendre pour garantir à l´enfant, une intervention adéquate, assurer une protection de ses droits surtout son droit au respect dans son intégrité physique et psychique.

### Etat des connaissances sur le sujet

- *Les agressions sexuelles aux enfants constituent un problème de santé publique souvent sous-déclaré ;*
- *Les enfants et les adolescents sont les plus à risque de subir ce type de violence; leur prévalence dépend des facteurs individuels, familiaux et sociétaux ;*
- *L'abus sexuel envers les enfants engendre de nombreux traumatismes; des signaux de détresse différents peuvent être émis par le mineur et qui devraient servir d'appels au diagnostic*.

### Contribution de notre étude à la connaissance

- *Nous avons pu identifier à travers notre enquête, un profil épidémiologique des enfants victimes de violence sexuelle ce qui permettrait d´adapter les moyens d´intervention de prise en charge et de prévention;*
- *Nous avons pu constater à travers notre enquête que la symptomatologie relevée chez les enfants victimes de violence sexuelle est riche, la souffrance est exprimée de différentes manières;*
- *Il faut disposer de stratégies qui visent à amoindrir la vulnérabilité des enfants ou à renforcer leur sécurité, afin de réduire la probabilité de survenu des violences sexuelles*.
